# Systematic review of bilateral epididymal leiomyomas

**DOI:** 10.1308/rcsann.2025.0022

**Published:** 2025-07-24

**Authors:** KH Pang, M Walkden, A Haider, P Sangster, HM Alnajjar, A Muneer, WG Lee

**Affiliations:** ^1^ University College London Hospitals NHS Foundation Trust, UK; ^2^ University College London, UK; ^3^ Chelsea and Westminster Hospital NHS Foundation Trust, UK; ^4^ St Peter's Andrology Centre, UK; ^5^ NIHR Biomedical Research Centre, University College London Hospitals NHS Foundation Trust, UK

**Keywords:** Intrascrotal, Paratesticular, Epididymis, Leiomyoma, Tumour

## Abstract

**Purpose:**

Epididymal leiomyomas (LM) are rare benign tumours. Bilateral LM are even more uncommon and there are no management guidelines on LM. We performed a systematic review to update the number of epididymal LM cases reported, and to summarise how these tumours have been managed at different centres with a description of our own experience.

**Method:**

The systematic review was performed according to the PRISMA guidelines. The PubMed database was searched for articles on epididymal LM. Data extracted included patients’ age, presenting complaint, diagnostic tests, management and follow-up. In addition, a case from our own institution was presented.

**Results:**

The systematic search identified 120 articles of which 29 articles including 32 patients were analysed. There were 27 (84.4%) unilateral cases and 5 (15.6%) bilateral cases. Surgical treatments included lesion excision, *n *= 14 (43.8%); orchidectomy, *n *= 10 (31.3%); partial epididymectomy, *n *= 5 (15.6%); and total epididymectomy, *n *= 3 (9.4%). Final histology revealed 21 LM (65.6%) and 11 leiomyoadenomatoid tumours (34.4%). At a median (interquartile range) follow-up of 14 (8–12) months, there were no cases of recurrence. Our patient, a 53-year-old man, presented with bilateral epididymal lesions for over 1 year and underwent ultrasound scan and positron emission tomography imaging. The imaging findings were indeterminate, hence an excisional biopsy on one side was performed which revealed an epididymal LM. Because LM are benign, further surgery on the contralateral side was not performed.

**Conclusion:**

Testis-sparing surgery appears to be feasible and safe, limiting the morbidity of radical orchidectomy. Because epididymal LM are rare, a multidisciplinary assessment and management are advised.

## Introduction

Paratesticular tumours, especially tumours of the epididymis, are rare. Epididymal tumours represent around 5% of all intrascrotal tumours and the majority (∼75%) are benign.^1^ Adenomatoid tumours are the most common tumours of the epididymis, representing around one-third of all paratesticular tumours. Leiomyomas (LM; 6%) are the second most common epididymal tumours.^2,3^ LM are benign tumours arising from mesenchymal cells responsible for smooth muscle formation, and patients usually present in their fifth decade of life.^4^ LM can occur alone or mixed with adenomatoid components – leiomyoadenomatoid (LMA) tumours. Epididymal LM presenting bilaterally are extremely rare and fewer than ten cases have been reported in the literature. Moreover, there are no guidelines on the management of these tumours. A systematic review was performed to estimate the number of LM cases reported and to summarise how these rare tumours have been managed at different centres.

## Methods

A systematic search was performed with reference to the PRISMA checklist^5^ to summarise how these tumours are managed by other centres (Supplementary Table 1 – available online). A search of the PubMed database was performed on 10 March 2024 using the terms, (leiomyoma) AND (epididymis). All manuscripts reporting unilateral or bilateral epididymal LM were included for abstract screening. Non-English articles were excluded. An up-to-date search was performed on 13 September 2024 and no further manuscripts were identified for inclusion.

Abstract and full-text articles were screened by KHP and WGL, any conflicts were resolved between the two abstract screeners. Data extracted (KHP) included, age of the patient, presenting complaint, duration of symptoms, imaging modality used and its characteristics, surgical intervention, histopathology, and follow-up. A narrative synthesis was undertaken due to significant heterogeneity.

## Findings

**Figure 1:**
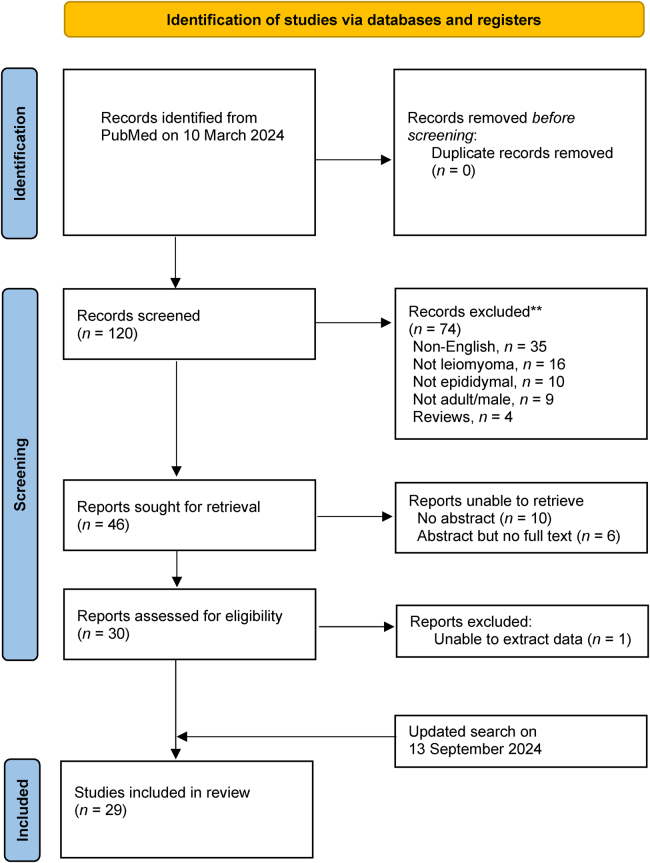
PRISMA flow chart

Overall, 120 titles and abstracts were retrieved from the initial search. Following abstract and full-text articles screening, 29 articles were included for analysis^6–34^ (Figure 1), consisting of 32 patients with a median (interquartile range [IQR]) age of 53 (47–59) years (Table 1). Most reports were from Asia (*n *= 12, 41.4%), followed by the United States (*n *= 9, 31.0%) and Europe (*n *= 8, 27.6%).

**Table 1:** Characteristics of the epididymal leiomyoma and leiomyoadenomatoid cases identified from the systematic search

Author	Year	Country	Age (years)	Presenting complaint	Duration symptoms (months)	USS features	MRI features	Tumour side	Tumour size (mm)	Treatment	Histology	Follow-up (months)
Krishnamurthy *et al* (34)	2023	USA	49	Mass	NR	Heterogeneous	Non-enhancing	Right	14	Orchidectomy	LMA	NR
			66	Mass	NR	Heterogeneous, peripheral vascularity	Not performed	Right	21	Partial epididymectomy	LMA	NR
Elyaguov *et al* (6)	2022	USA	58	Painful mass	36	Heterogeneous, echogenic mass, central vascularity	Not performed	Right	37	Excision	LMA	NR
Lucianò *et al* (7)	2022	Italy	44	Painless mass	48	Vascularity	Not performed	Right	35	FSE (adenomatoid) and excision	LMA	3
Hao *et al* (8)	2022	China	74	Painless, enlarging mass	276	Hypoechoic, heterogeneous	Not performed	Bilateral	106, 96	Excision	LM	NR
Almohaya *et al* (9)	2021	Saudi Arabia	49	Painless non-enlarging mass	60	Hyperechoic, internal vascularity	Hypointense on T2	Right	23	Excision	LMA	NR
Wazwaz *et al* (10)	2020	Qatar	33	Painless, enlarging mass	24	Hypoechoic, heterogeneous, internal vascularity	Low T2 signal	Left	13	Excision	LMA	NR
Shehabeldin *et al* (11)	2020	USA	28	Painless, enlarging mass	NR	Hypoechoic	Not performed	Right	10	Radical orchidectomy	LMA	14
			50	Painless, enlarging mass	60	Heterogeneous	Not performed	Right	30	Radical orchidectomy	LMA	22
Dell’Aversana *et al* (12)	2019	Italy	57	Painless mass	NR	Regular margins, heterogeneous, no vascularity	Hypointense on T2, isointense on T1, contrast enhancement	Bilateral	45, 55	Excision	LM	NR
Raghavendran *et al* (13)	2017	India	59	Painless non-enlarging mass	24	Hyperechoic	Not performed	Unilateral	60	FNAC (spindle cells) and excision	LM	24
Marcou *et al* (14)	2017	Germany	50	Painless, enlarging mass	60	Heterogeneous	Not performed	Left	20	Excision	LM	6
Fernandez *et al* (15)	2017	India	56	Painless mass	8	NR	Not performed	Bilateral	30	Excision	LM	NR
Cazorla *et al* (16)	2014	France	57	Painless non-enlarging mass	"Several" years	Hypoechoic, heterogeneous	Not performed	Right	20	FSE and excision	LMA	12
Cakiroglu *et al* (17)	2014	Turkey	47	Painless mass	2	Hypoechoic, vascularity	Not performed	Right	30	FSE (spindle cells) and partial epididymectomy	LM	36
Arpali and Tok (18)	2013	Turkey	42	Mass and acute pain	Mass (12), pain (4h)	Mass and torsion	Not performed	Left	40	Radical orchidectomy	LM	12
Yeh *et al* (19)	2006	Taiwan	53	Painless, enlarging mass	48	Hypoechoic, internal vascularity	Not performed	Left	20	Excision	LM	15
Fumo *et al* (20)	2006	USA	56	Painless mass	NR	Hypoechoic, internal vascularity	Not performed	Left	16	FSE (spindle cells) and partial epididymectomy	LM	36
Kikugawa *et al* (21)	2003	Japan	76	Mass	NR	NR	Not performed	Left	15	Epididymectomy	LM	22
Yilmaz *et al* (22)	2002	Turkey	68	Painless mass	NR	Hypoechoic, homogeneous	Not performed	Right	32	Orchidectomy	LM	2days
Kausch *et al* (23)	2002	Germany	44	Painful mass	2	Combined hypodense and hyperdense	Not performed	Right	30	FSE (LM) and epididymectomy	LMA	8
Yusim *et al* (24)	2001	Israel	55	Mass	5	NR	Not performed	Right	20	Partial epididymectomy	LM	48
Bozlu *et al* (25)	2000	Turkey	53	Painless mass	24	Heterogeneous	Not performed	Right	35	FSE (benign) and epididymectomy	LM	22
Hertzberg *et al* (26)	1996	USA	64	Painless mass	>60	Hypoechoic	Not performed	Left	60	Radical orchidectomy	LM	NR
Block and Block (27)	1995	USA	41	Painful mass	12	Hypoechoic, heterogeneous	Not performed	Left	36	Radical orchidectomy	LM	12
Leonhardt and Gooding (28)	1993	USA	72	Painless mass	NR	Heterogeneous, calcification	Not performed	Bilateral	40 and 20	Left-sided excision (Right-side not excised)	LM	NR
Payan *et al* (29)	1967	USA	55	Hydrocele	60	Hydrocele	Not performed	Right	NR	Orchidectomy	LM	"Several" months
			70	Hydrocele	120	Not performed	Not performed	Left	NR	Orchidectomy	LM	"Several" months
Jablokow and Meyer (30)	1957	USA	59	Painless, enlarging mass	120	Not performed	Not performed	Left	50	FSE (LM) and partial epididymectomy	LM	6 days
Henderson (31)	1956	UK	47	Mass	6	Not performed	Not performed	Bilateral	15 and 6	Excision	LM	8
Laird (32)	1953	UK	31	Mass	10	Not performed	Not performed	Right	110	Radical orchidectomy	LM	NR
Wilson (33)	1949	UK	46	Painless mass	3	Not performed	Not performed	Left	15	Excision	LMA	NR
			Median (IQR), 53 (47–59)		Median (IQR), 24 (9–60)			Bilateral, *n* = 5 Unilateral, *n* = 27	Median (IQR), 30 20–40)			Median (IQR), 14 (8–22)

FNAC = fine needle aspiration cytology; FSE = frozen section examination; IQR = interquartile range; LM = leiomyoma; LMA = leiomyoadenomatoid; MRI = magnetic resonance imaging; NR = not reported; USS = ultrasound scan

Most patients (93.8%) presented with a palpable mass, but two presented with hydrocoeles and were later found to have epididymal LM when they were re-explored for persistent pain.^29^ One patient presented with acute pain and testicular torsion.^18^ The median (IQR) duration of symptoms was 24 (9–60) months. The majority (84.4%) had a scrotal ultrasound scan (USS) to evaluate their mass and 3 (9.4%) men underwent magnetic resonance imaging (MRI) as well for further characterisation. USS findings were not consistent, whereby some lesions were reported to be hypoechoic or hyperechoic, with or without internal vascularity (Table 1). Overall, five (15.6%) patients had bilateral tumours, and the median (IQR) tumour size was 30 (20–40)mm. Surgical treatment included excision of the lesion, *n *= 14 (43.8%); orchidectomy, *n *= 10 (31.3%); partial epididymectomy, *n *= 5 (15.6%); and total epididymectomy, *n *= 3 (9.4%) (Table 1). A frozen section examination (FSE) was performed in seven (21.9%) patients and a fine needle aspiration cytology (FNAC) was performed in one (3.1%) patient confirming a benign tumour (Table 1). Final histology revealed 21 LM (65.6%) and 11 LMA (34.4%). At a median (IQR) follow-up of 14 (8–12) months, there were no cases of recurrence or malignant transformation.

## Case report

**Figure 2:**
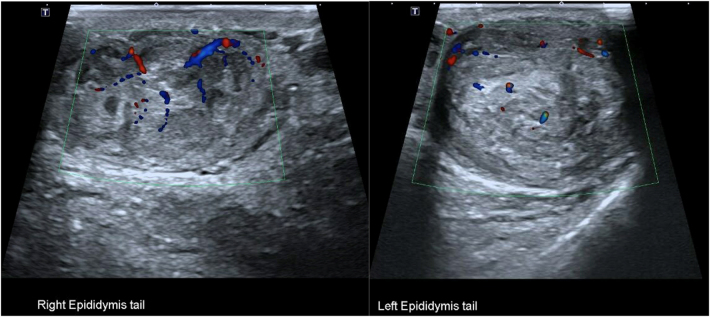
Ultrasound image of the epididymal tail lesions. The lesions could be located as arising from the epididymal tails and appearances were identical bilaterally. The lesions were well-defined and of mixed echogenicity with mild internal vascularity. No calcification or convincing whirl pattern was seen.

**Figure 3:**
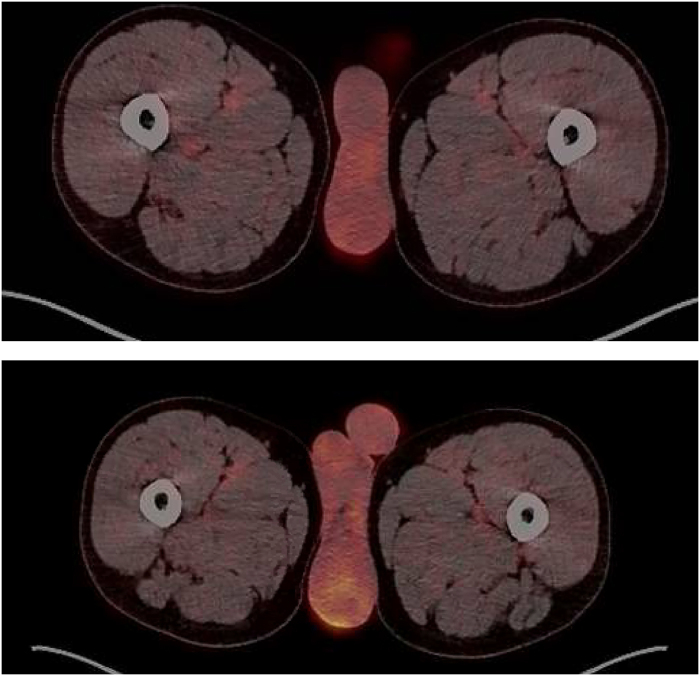
Positron emission tomography–computed tomography image of the scrotum: (a) normal very low-level physiological uptake in the testicles bilaterally; (b) increased uptake in the left epididymal tail within the leiomyoma.

**Figure 4:**
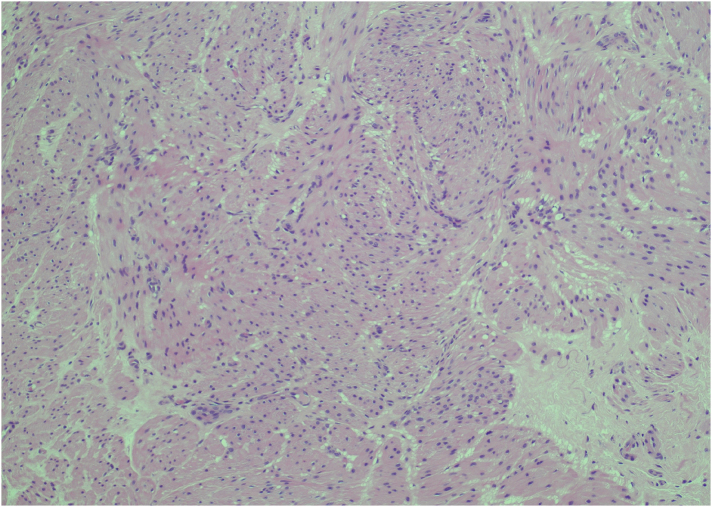
Histopathological slide of epididymal leiomyoma. Magnification ×10. Haematoxylin and eosin stain. Slide demonstrating interlacing bundles of bland smooth muscle cells.

Here, we present the management of a similar case in our institution. A 53-year-old man was referred for painless scrotal swelling over 15 months that was slowly enlarging. There was no history of trauma, and he worked as a roofer. On examination, both testes were palpable and had a normal size and texture. Superiorly there were bilateral hard, smooth, non-tender epididymal masses. No lymphadenopathy or any other abnormalities were identified on clinical examination. A scrotal USS demonstrated bilateral solid lesions measuring 62×57×37mm (volume 68.6ml) on the left and 42×36×27mm (volume 20.8ml) on the right. These could be seen as separate from the testicle and could be located as arising from the epididymal tails. They were of mixed echogenicity and did not contain any calcification. Doppler examination demonstrated definite internal vascularity each with two to three vessels, but the lesions were not hypervascular (Figure 2). The paratesticular location and their presence bilaterally suggested that the lesions were likely to be either benign or representing a systemic process. An interval USS was repeated after 6 weeks showing no increase in the size of the lesions. It was noted that the patient’s father may have had pulmonary sarcoidosis, and therefore this was initially explored as a differential diagnosis, but a subsequent chest x-ray was clear. He was referred to rheumatology for further assessment. There was no history of shortness of breath, cough, dyspnoea, rash, recent infective or systemic illness. Further investigations were requested for sarcoidosis, which included bloods and a positron emission tomography–computed tomography (PET–CT) scan. Bloods for C reactive protein, erythrocyte sedimentation rate, immunoglobulins and QuantiFERON gold were normal. The PET–CT scan showed normal physiological uptake in the testes with increased uptake in the left epididymis and to a lesser extent the right epididymis (Figure 3). PET uptake has been shown to be increased in cases of epididymal smooth muscle tumours.^35^ No extra-scrotal site for sarcoidosis or malignancy was found. The patient was discussed at a specialist multidisciplinary team (sMDT) meeting and the differential diagnoses included a benign epididymal tumour such as an adenomatoid tumour, an epididymal LM or a papillary cystadenoma. The patient had not had a vasectomy, therefore sperm granulomas were discounted. A malignant process such as epididymal metastasis or leukaemia was felt unlikely, but an epididymal leiomyosarcoma was felt to be a possibility. Lastly an inflammatory process such as sarcoidosis or tuberculosis was considered, but had been excluded. The outcome of the sMDT was to offer the patient a biopsy of the lesion to obtain a histopathological diagnosis before considering any radical surgery. An excisional biopsy of the left-sided lesion was performed following delivery of the left testis through a subinguinal incision. Histopathology showed bundles of intersecting smooth muscle fibres with minimal oedema and chronic inflammation. A single mitotic figure was identified (Figure 4). The appearances were suggestive of an LM of the epididymis. Staining and tissue culture were negative for acid-fast bacilli. The patient’s postoperative course was uneventful.

The patient was re-discussed at the sMDT. Because LM are benign, the right-sided LM was not excised and the decision made to offer the patient a surveillance USS at 6 months, and discharge from follow-up if appearances remained stable. The 6-month interval USS showed no significant changes, and the patient was discharged back to his general practitioner.

## Discussion

The present case revealed a 53-year-old man with a 16-month history of bilateral scrotal swelling shown to be an epididymal LMs.

This systematic review of the literature for the first time confirmed the rarity of this benign tumour. Only 32 tumours have been reported in the epididymis, with bilateral tumours being rarer still (*n *= 5, 15.6%). Reassuringly, malignant transformation has not been described. There was significant heterogeneity in the reported investigation and management of LM and LMA. The median (IQR) age at presentation was 53 years (47–59), and duration of symptoms was 24 months (9–60). However, LM has been reported in a 28-year-old man^11^ and should be considered in the differential diagnosis for any epididymal lesion. The present patient underwent a slightly different diagnostic pathway compared with other reported cases. Sarcoidosis was initially suspected, but excluded following blood tests and PET–CT. A subsequent excisional biopsy demonstrated a LM.

LMs arise from smooth muscle and can be found in the kidney, bladder, spermatic cord, penis and the urethra.^24,36^ LM may have an adenomatoid component (LMA tumours) that comprised 34.4% of reported cases. LM are slow-growing tumours, and asymptomatic patients may present up to two decades from when they first noticed the mass.^8^ Although patients commonly present with a painless mass, one patient presented with acute pain for 4h and was found to have testicular torsion on scrotal exploration.^18^ In addition, two patients had surgery for hydrocoeles, but were found to have epididymal LM when they were re-explored for persistent pain.^29^ The present case had a tumour with a maximum diameter of 62mm, which is in the higher range compared with tumours identified in our search. There were two cases in which tumour sizes were over 100mm representing the largest tumours reported in the literature.^8,32^

Investigations undertaken prior to diagnosis included tumour markers and ultrasonography. Tumour markers were invariably negative. On USS, our patient had lesions with identical findings. They were of mixed echogenicity and had definite internal vascularity. They did not contain calcification, nor did they demonstrate an obvious whirling pattern. Other reports in the literature demonstrated heterogeneous appearances with a mixture of hypo- or hyper-echogenicity. Internal vascularity may be present and absent. Calcification if seen is described as punctate. A more specific finding for LM is the ‘whirling pattern’ that is characterised by alternating linear areas of high and low echogenicity. These sonographic findings come from the histological features of interlacing bundles of smooth muscle cells in these tumours.

MRI can be used as a problem-solving tool in the scrotum. MRI is best placed to identify internal vascularity and the exact anatomical origin of a lesion. It was non-contributory in the present case because both had been correctly identified by USS. An MRI can also detect fat and can give information on cellularity. In this case, it was felt that ultimately imaging would not be able to confidently differentiate a benign from a malignant process and therefore a tissue diagnosis was needed, especially given the slightly unusually large size of the lesions.

The goal of surgical management is twofold: first, to establish a histological diagnosis and second, to excise the lesion, if required. The systematic review identified 14 patients who had an excisional biopsy, all revealed LM and therefore epididymectomy or orchidectomy was not performed. A total of ten (31.3%) patients had an upfront orchidectomy, five (15.6%) had a partial epididymectomy and three (9.4%) had a total epididymectomy.

The preferred pathway at our centre is to perform organ-sparing surgery in the form of excisional biopsies, rather than epididymectomy or orchidectomy, to preserve some testicular function. Alternative options are to perform FSE or FNAC and to proceed to more radical surgery if the histopathology revealed cancer. In the literature, FSE and FNAC were performed in seven (21.9%) and one (3.1%) cases, respectively. All specimens revealed no cancer, and no further surgery was performed. However, FSE is not always available and requires experienced histopathologists to examine the specimen since LM and LMA are rare tumours.

Bilateral cases are very rare with only five cases reported in the literature. Consistent with the present approach, Leonhardt and Gooding^28^ performed an excision biopsy on one side confirming a LM and further contralateral surgery was not performed. This approach may preserve fertility by not violating the contralateral epididymis or testis. Even limited excision biopsy may lead to epididymal scarring and subsequent ipsilateral epididymal obstruction.^37^ By contrast, upfront complete excision of the mass was performed on both sides in the other four cases. The current experience of the literature reported no cases of malignant transformation with a median (IQR) follow-up of 14 (8–12) months. Therefore, preserving the contralateral lesion may be appropriate. Complete excision was offered to the present patient but he declined further intervention as he was asymptomatic.

### Study limitations

Our search identified 120 articles, but 35 articles were excluded because they were not in English. Furthermore, 46 articles could have been included for data analysis following abstract screening, but 16 of them could not be retrieved. Therefore, there may be more epididymal LM cases reported in the literature than the 29 articles we retrieved for analysis.

## Conclusion

The narrative synthesis of the data suggests that an organ-sparing approach with FNAC or excision biopsy (with or without FSE) may be a safe approach with the aim of limiting surgical morbidity. Multidisciplinary assessment and management are strongly recommended given the rarity of malignant epididymal tumours.

## Ethics approval and informed consent

Patient consent was obtained for this review.

## Data availability statement

All data are included in this manuscript.
